# Poster Session I - A23 FASTING REMODELS THE COLONIC MICROBIOTA AND INCREASES GOBLET CELL AUTOPHAGY TO PRESERVE THE MUCUS BARRIER

**DOI:** 10.1093/jcag/gwaf042.023

**Published:** 2026-02-13

**Authors:** S G Sorboni, L S Celiberto, H Yang, H Sham, B Vallance

**Affiliations:** The University of British Columbia, Vancouver, BC, Canada; The University of British Columbia, Vancouver, BC, Canada; The University of British Columbia, Vancouver, BC, Canada; The University of British Columbia, Vancouver, BC, Canada; The University of British Columbia, Vancouver, BC, Canada

## Abstract

**Background:**

Fasting induces major metabolic changes, yet its effects on the gut microbiota and mucus barrier remain unclear. In the absence of dietary nutrients, intestinal microbes degrade host-derived mucin O-glycans, potentially compromising barrier integrity. Understanding how the colon preserves mucosal protection during nutrient stress may reveal adaptive host–microbiota mechanisms.

**Aims:**

To define how acute fasting remodels colonic microbial metabolism and epithelial responses that sustain the mucus barrier.

**Methods:**

Female C57BL/6J mice (6–8 weeks) were fasted for 48 h with water ad libitum. Systemic metabolism was evaluated by measuring body weight, glucose, and β-hydroxybutyrate. Microbiota composition and function were analyzed using 16S rRNA sequencing, absolute quantification, and shotgun metagenomics. Short-chain fatty acids (SCFAs) were quantified by LC-MS. Colonic mucus structure and microbial localization were assessed by histology, lectin staining, and FISH, while LC3 immunostaining measured goblet cell autophagy.

**Results:**

Fasting caused ∼20% body-weight loss, elevated β-hydroxybutyrate (>2 mmol/L), and reduced blood glucose (<60 mg/dL), indicating a systemic shift toward ketone metabolism. Concurrently, it reshaped the gut microbiota: the mucin-degrading *Akkermansia muciniphila* expanded ∼100-fold, whereas SCFA-producing Firmicutes and Clostridia declined, reducing luminal SCFAs by ∼80%. Metagenomic profiling showed enrichment of mucin-targeting CAZymes, reflecting enhanced microbial capacity for mucin O-glycan utilization. Despite this mucolytic shift, histology and lectin staining revealed a modestly thicker inner mucus layer and expanded distal mucus sublayer, suggesting an adaptive reinforcement of microbial exclusion. LC3 staining demonstrated elevated goblet-cell autophagy, consistent with secretory autophagy recycling mucin granules to sustain mucus secretion under nutrient limitation. Collectively, these findings indicate that fasting triggers a coordinated host–microbiota response in which autophagy-driven mucus renewal maintains epithelial protection and microbial segregation during nutrient stress.

**Conclusions:**

Fasting drives ecological and metabolic remodelling of the colonic microbiota, while also yet preservinges mucosal protection through an autophagy-dependent goblet cell response that sustains mucus secretion under nutrient stress. This adaptive response maintains continuous mucus renewal and prevents barrier depletion despite reduced SCFA levels and increased mucin degradation, thereby maintaining spatial segregation between the microbiota and epithelium during fasting. These findings uncover a host-adaptive mechanism that maintains mucus barrier integrity and highlight how fasting dynamically reshapes microbiota-host interactions to preserve mucosal homeostasis.

**Funding Agencies:**

NoneNSERC

